# Definitions of, Advances in, and Treatment Strategies for Breast Cancer Oligometastasis

**DOI:** 10.3390/cancers17142406

**Published:** 2025-07-21

**Authors:** Tadahiko Shien, Shogo Nakamoto, Yuki Fujiwara, Maya Kosaka, Yuki Narahara, Kento Fujii, Reina Maeda, Shutaro Kato, Asuka Mimata, Ryo Yoshioka, Chihiro Kuwahara, Takahiro Tsukioki, Yuko Takahashi, Tsuguo Iwatani, Maki Tanioka

**Affiliations:** Department of Breast and Endocrine Surgery, Okayama University Hospital, Okayama 7008558, Japan; p92c9f20@s.okayama-u.ac.jp (S.N.); pafv9w6o@okayama-u.ac.jp (Y.F.); pt2b11tg@okayama-u.ac.jp (M.K.); racheljasmin2214259@icloud.com (Y.N.); pa1f4koe@okayama-u.ac.jp (K.F.); pxkh7mnv@okayama-u.ac.jp (R.M.); p6qh32jj@okayama-u.ac.jp (S.K.); p59483pq@s.okayama-u.ac.jp (A.M.); yoshiokar0907@gmail.com (R.Y.); me422107@s.okayama-u.ac.jp (C.K.); takahirot5974@yahoo.co.jp (T.T.); yukotaka@okayama-u.ac.jp (Y.T.); t.iwatani@marianna-u.ac.jp (T.I.); pdq249jj@s.okayama-u.ac.jp (M.T.)

**Keywords:** oligo-recurrence, breast cancer, local therapy

## Abstract

Oligometastasis represents a clinically relevant state of limited metastatic disease that could be amenable to selected local therapies in carefully chosen patients. Although initial trials such as SABR-COMET demonstrated a survival benefit with aggressive local treatment, breast cancer was underrepresented. Subsequent breast cancer-specific trials, including NRG-BR002, failed to show a clear survival benefit, highlighting uncertainties and the need for further refinement in patient selection and integration with systemic approaches. The definitions of oligometastasis continue to evolve, incorporating radiological, clinical, and biological features. Advances in imaging and molecular profiling suggest that oligometastatic breast cancer might represent a distinct biological subtype, with potential biomarkers including PIK3CA mutations and YAP/TAZ expression. Organ-specific strategies using stereotactic radiotherapy, surgery, and proton therapy have shown favorable local control in certain settings, though their impact on the overall survival remains under investigation. Emerging techniques, including circulating tumor DNA (ctDNA) analysis, are being explored to improve patient selection and disease monitoring. Ongoing trials may provide further insight into the role of local therapy, particularly in hormone receptor-positive or HER2-positive subtypes. Local and systemic strategies need to be carefully coordinated to optimize the outcomes. This review summarizes the current definitions of and evidence and therapeutic considerations for oligometastatic breast cancer and outlines potential future directions.

## Simple Summary

Oligometastatic breast cancer is a limited metastatic state in which selected patients may benefit from local therapies such as surgery, stereotactic radiotherapy, or proton therapy. While early trials like SABR-COMET suggested potential survival benefits with local treatment, breast cancer-specific studies such as NRG-BR002 have not demonstrated clear improvements, highlighting the importance of appropriate patient selection and standardized treatment strategies. The definition of oligometastasis is still evolving, incorporating radiological, clinical, and biological factors. Advances in imaging and molecular profiling suggest that oligometastatic disease may represent a distinct biological subtype, with candidate biomarkers including PIK3CA mutations and YAP/TAZ expression. Liquid biopsy tools, such as circulating tumor DNA analysis, are also being investigated for their potential to guide treatment decisions. Ongoing clinical trials are expected to clarify the role of local therapy, especially in hormone receptor-positive and HER2-positive subtypes. Local and systemic therapies must be carefully coordinated to achieve long-term disease control.

## 1. Background: Advances Leading to the Focus on Oligometastasis

Oligometastasis, first proposed by Hellman and Weichselbaum in 1995, describes an intermediate state of cancer spread where metastases are limited and potentially curable using local therapies [1,2]. Advances in treatment modalities such as stereotactic body radiotherapy (SBRT), proton beam therapy (PBT), and systemic therapies have made this concept increasingly relevant. In addition, improved diagnostic tools, particularly PET-CT and MRI, have enabled the more precise detection of small metastases, facilitating early intervention [2,3]. While PET-CT plays a pivotal role in the early detection of oligometastasis due to its high sensitivity, it is not yet a routine diagnostic tool used in everyday clinical practice. The concept underlying discussions of oligometastasis often revolves around controlling small lesions detected exclusively by PET-CT through SBRT or surgery, with the expectation that this approach will improve the patient outcomes. However, the potential for lead time bias should be considered when interpreting these findings. Lesions only detected by PET-CT may improve the prognosis because their detection and treatment preceded the point at which they would have been identified using standard CT. This temporal advantage could give the impression of prolonged survival without reflecting an actual improvement in the outcomes. Thus, the utility of PET-CT in this context must be evaluated with careful attention to these biases.

Clinical trials like SABR-COMET demonstrated that comprehensive metastasis-directed therapy improves the progression-free survival and overall survival (OS), highlighting the potential for long-term control or a cure in selected cases [4,5]. These trials proved that targeted interventions for limited metastatic disease can extend survival compared to standard palliative care. The robustness of the findings is supported by well-defined patient cohorts and rigorous trial designs, underscoring their clinical relevance. Additional studies, such as the EA2108 trial, explored the role of early local therapy in stage IV breast cancer but found no significant survival benefit when local therapies were added to systemic treatment [6].

SABR-COMET demonstrated that aggressive local therapies applied to metastatic sites could improve the outcomes; however, breast cancer cases were underrepresented in this study. Subsequently, the NRG-BR002 trial focused exclusively on the role of local therapies in treating breast cancer oligometastasis [7]. Contrary to SABR-COMET, this trial did not demonstrate a survival benefit from local therapies. Notably, the NRG-BR002 trial did not mandate the use of advanced imaging techniques such as PET/CT for metastatic diagnosis, nor did it standardize the systemic therapy regimens. Subgroup analyses revealed that local therapies did not improve the outcomes even in cases with favorable prognoses, such as single metastases, and often led to new lesion development. Additionally, triple-negative breast cancer (TNBC) cases showed poorer outcomes in the local therapy group. These findings underscore the necessity of integrating effective systemic therapies before and after local interventions to achieve optimal results. These results suggest that the study design and treatment strategies used in NRG-BR002 may not have been optimal. Notably, the increased incidence of new metastatic lesions in the local therapy arm raises questions about the adequacy of pre-treatment imaging, such as PET-CT, in accurately staging the disease. Furthermore, it remains unclear whether effective systemic therapy was consistently continued after local treatment. These factors may have contributed to the lack of benefits observed in the trial, emphasizing the importance of using standardized imaging protocols and systemic therapy regimens in future studies.

## 2. Definitions of Oligometastasis

### 2.1. Clinical and Radiological Criteria

Oligometastasis is typically defined by the presence of up to five metastatic lesions confined to specific anatomical regions and amenable to local therapy [1,6]. Both references [1] and [6] provide foundational criteria, with Hellman et al. offering an initial definition and elaborating on this classification’s clinical applications and limits. For instance, the ESTRO and EORTC criteria emphasize the importance of the clinical course and number of lesions in defining this state [1].

### 2.2. Biological Underpinnings

The idea behind local treatment for oligometastasis is that early intervention to reduce the tumor burden can create a near-curative state, thereby improving the prognosis. However, evidence from ovarian cancer studies challenges this assumption. For instance, in ovarian cancer, initiating treatment based solely on elevated CA125 levels (an average of six months earlier) versus starting treatment after tumor detection in CT scans showed no difference in the outcomes [8]. Additionally, despite the frequent use of second cytoreductive surgery in ovarian cancer, it has not consistently demonstrated a survival benefit. These findings raise questions about the rationale for using local treatments in oligometastatic breast cancer. The differences in the tumor biology between these cancers may play a significant role in the efficacy of regional interventions. This underscores the importance of considering both the tumor burden and biological characteristics when evaluating treatment strategies for oligometastasis. Future studies should focus on integrating these aspects to develop novel approaches tailored to the unique biology of oligometastatic disease. Emerging research suggests that oligometastasis may represent a biologically distinct entity. Mutations such as PIK3CA H1047R and the overexpression of markers like YAP/TAZ are associated with oligometastatic breast cancer (OMBC) and may predict better responses to local therapy [9].

## 3. Treatment Strategies for Oligometastasis

### 3.1. Importance of Local Therapies

Local therapies like SBRT, surgical resection, and radiofrequency ablation (RFA) are critical in treating oligometastasis. These interventions aim to achieve high local control rates and improve the OS when integrated with systemic therapy. Hellman et al. established the foundational role of local treatments in managing limited metastases, while reference [10] provides evidence of their impact on the survival outcomes across different clinical scenarios.

SBRT is particularly effective for small metastatic lesions in the brain, lung, and liver, achieving local control rates of up to 90% in specific cohorts [2,10]. Proton beam therapy (PBT) has shown promise in treating liver oligometastasis, providing high local control rates with minimal toxicity [11]. Surgical resection remains the gold standard for specific sites, such as lung metastases, where complete resection is feasible [6,10]. Trials like MF07-01 have demonstrated improved survival outcomes in selected patients undergoing primary tumor resection in stage IV breast cancer [5,6].

### 3.2. Primary Tumor Resection in Stage IV Cancer

Several randomized trials have evaluated primary tumor resection in patients with de novo stage IV breast cancer. While the EA2108 trial showed no significant survival benefit with the addition of primary tumor resection to systemic therapy, it highlighted the importance of systemic control [6]. Conversely, the MF07-01 trial demonstrated a survival advantage in selected patients undergoing surgery, particularly those with a limited metastatic burden and good performance status [5,6].

However, it is essential to note that most prospective trials have not indicated an overall survival (OS) benefit for primary tumor resection, with its utility being limited to improving the local control [11]. One key consideration is that local therapy for the primary tumor may exacerbate distant metastases. This phenomenon may result from delays in systemic therapy during the local treatment period or the release of cytokines that stimulate the growth of the remaining metastases.

Therefore, it is crucial to integrate effective systemic therapies alongside or following local treatment. Subgroup analyses from trials suggest that primary tumor resection may benefit patients with isolated bone metastases or limited single-organ metastases, suggesting the possibility that such surgical interventions could be effective for oligometastatic disease [11].

## 4. Organ-Specific Strategies for Treating Oligometastasis

### 4.1. Liver

The liver is a common site for oligometastasis, particularly in colorectal and breast cancer. SBRT and PBT offer effective, minimally invasive options with local control rates exceeding 90% [12]. However, liver metastases are associated with a poorer prognosis compared to other oligometastatic sites, and local therapies may have a limited impact on the long-term outcomes. Surgical resection, which is often minimally invasive when performed using modern techniques, remains a critical strategy for the treatment of selected cases, particularly when histological confirmation is needed. Recent evidence has also explored the potential role of liver transplantation in the treatment of selected patients with favorable tumor biology [13,14].

Proton beam therapy (PBT) has shown high local control rates in liver metastases from breast cancer, as reported in a nationwide Japanese cohort, with three-year local control rates of 100% [15,16]. However, further research is needed to establish the role of PBT in treatment compared to that of other modalities. SBRT remains less commonly used in liver metastases, as its associated risks include radiation-induced liver disease and it has limitations in treating larger tumors [16,17]

### 4.2. Lung

Lung oligometastases are amenable to surgical resection and SBRT, though the optimal approach depends on the individual patient and lesion characteristics. Surgical resection is particularly advantageous when the histopathological confirmation of metastasis is required to distinguish between primary lung cancer and metastatic lesions, which can occur in up to 12–57% of cases [14]. Video-assisted thoracoscopic surgery (VATS) has improved the safety of lung resections and made them minimally invasive, with no perioperative complications reported in recent cohort studies.

Moreover, patients who underwent metastasectomy demonstrated better progression-free survival (PFS) than those who did not, emphasizing the potential curative benefit in selected cases [15]. However, SBRT provides a non-invasive alternative with high local control rates but carries risks such as radiation pneumonitis, which can complicate post-treatment evaluations and affect pulmonary function [16]. Retrospective analyses and ongoing trials are aiming to determine the optimal patient population for treatment using these modalities.

### 4.3. Brain

For brain metastases, local therapies are considered the standard of care, particularly in patients presenting with up to four lesions. Fractionated stereotactic radiotherapy (SRT) is commonly employed to minimize the neurotoxicity while maintaining high local control rates [17]. The early detection of brain metastases using MRI followed by SRT is critical for symptom control and improved outcomes [18]. However, systemic therapies targeting specific subtypes of breast cancer, such as HER2-positive and triple-negative types, are emerging as valuable adjuncts or alternatives to local treatment [19].

Trastuzumab deruxtecan has demonstrated promising results in HER2-positive brain metastases, achieving progression-free survival rates of 14–17 months and overall survival rates exceeding 24 months in specific cohorts [3]. Future studies may further determine the optimal balance between systemic and local therapies for the management of brain oligometastases.

### 4.4. Bone

Bone metastases have traditionally been managed with radiotherapy, given its efficacy in pain relief and local control. SBRT is particularly effective in isolated bone metastases, achieving durable local control with minimal toxicity [20]. However, distinguishing oligometastatic from widespread bone involvement remains challenging, necessitating careful imaging and biopsies for accurate assessment [21]. While SBRT’s role in preserving quality of life is well-documented, its impact on the overall survival requires further investigation [12].

### 4.5. Lymph Nodes

Distant lymph node metastases, particularly contralateral axillary lymph node metastases (CAMs), may represent the most curable subset of oligometastases. Recent studies have suggested that CAMs should be regarded as oligometastatic-like disease, with aggressive local therapies potentially leading to long-term remission [10,22]. Cases involving CAMs with no additional visceral metastases may benefit significantly from surgical resection combined with systemic treatment, as these approaches have demonstrated improved progression-free and overall survival outcomes in selected patients.

## 5. Ongoing Studies

Several phase 3 trials are underway worldwide to evaluate the impact of local therapies on improving the outcomes in oligometastatic patients (Table 1). Many of these studies use the progression-free survival (PFS) as the primary endpoint; however, JCOG2110 stands out as the only trial utilizing the overall survival (OS) as its primary endpoint.

SBRT is the predominant intervention in these trials, with surgery reserved for cases where SBRT is not feasible. Notably, specific trials specifically include triple-negative breast cancer (TNBC) as a subgroup, but based on the findings from the NRG-BR002 study, the efficacy of local therapy in TNBC remains questionable. On the other hand, future research focusing on ER-positive or HER2-positive subtypes is eagerly awaited.

The results of these ongoing prospective trials are anticipated to clarify the indications for and optimal interventions regarding local therapies in oligometastasis. Detailed evaluations of these outcomes will provide significant insights into refining the treatment strategies for this unique metastatic state.

## 6. Future Directions

A significant challenge in oligometastatic disease is the lack of a universally accepted definition. While the number and size of metastases are essential for determining the feasibility of local therapy, they may not necessarily predict the long-term outcomes or the potential for a curative outcome. This underscores the need for the use of biomarkers or molecular characteristics to better stratify oligometastatic disease [11,13]_._

To achieve curative outcomes in metastatic breast cancer, both detectable metastases and microscopic metastases must be controlled. Local therapies such as SBRT and surgery play a crucial role in addressing visible lesions, while systemic therapies are essential for eradicating undetectable microscopic metastases. Circulating tumor DNA (ctDNA) testing is emerging as a promising tool for detecting minimal residual disease, helping to stratify patients and guide treatment decisions [13,22].

If the size and number of metastases are critical, early detection becomes essential, necessitating changes in postoperative follow-up protocols. However, the current evidence suggests that intensive postoperative surveillance does not improve the outcomes, raising questions about the relationship between early detection and clinical benefits. Future research should focus on defining subgroups of patients who may benefit from aggressive monitoring and early intervention strategies [13].

Additionally, integrating systemic therapies with local treatments remains an area of active investigation. Advances in imaging, liquid biopsy, and molecular profiling could pave the way for personalized approaches to define the oligometastatic state better and improve the outcomes [13,22].

Liquid biopsy technologies, including those used for the analysis of circulating tumor DNA (ctDNA), circulating tumor cells (CTCs), and exosomes, are gaining attention as non-invasive tools for the real-time monitoring of tumor dynamics. In oligometastatic breast cancer, these techniques may offer a way to detect minimal residual disease (MRD), predict early recurrence, and guide treatment decisions more precisely. For instance, ctDNA analysis can be used to stratify patients for intensified systemic therapy or early local intervention before radiographic progression [23]. Additionally, the molecular profiling of CTCs or exosomal RNA may reveal emerging resistance mechanisms and actionable targets [24]. Integrating liquid biopsy into clinical workflows could refine surveillance strategies and enable truly personalized approaches to managing oligometastatic disease [25,26].

Although the clinical significance of local therapy in oligometastatic breast cancer remains controversial, it continues to be practiced in various real-world settings, particularly in selected patients with a limited disease burden. While randomized trials such as NRG-BR002 have not demonstrated a clear survival benefit, their subgroup analyses and complementary observational studies suggest that certain patient populations may still derive value from local approaches. At present, however, the amount of breast cancer-specific evidence remains limited, and no universally accepted treatment algorithm exists. Therefore, instead of presenting a comprehensive review, this article aims to provide a forward-looking perspective based on the current gaps and anticipated future developments. As the ongoing clinical trials mature and new biological insights emerge, a full systematic review will be warranted to better define the role of local therapy in this unique clinical context.

In addition to refining trial design, several critical questions remain regarding the future of local therapy’s clinical implementation in OMBC. First, while mutations such as PIK3CA H1047R are frequently observed in breast cancer and may indicate less aggressive disease biology, their integration into workflows to select patients for local therapy requires further prospective validation. Future studies should investigate whether PIK3CA-mutated tumors derive a greater benefit from local interventions and how liquid biopsy tools can aid in identifying such candidates non-invasively.

Second, the definition of “optimal” systemic therapy in the context of OMBC remains unclear. Systemic regimens must be tailored not only to the subtype (e.g., endocrine-based therapy for ER-positive disease, anti-HER2 agents for HER2-positive disease) but also to the timing relative to that of local therapy. Studies are needed to evaluate the ideal sequencing, intensity, and duration of systemic treatment used in conjunction with local modalities.

Third, rather than relying solely on the lesion count, OMBC classification should evolve to incorporate biological characteristics—particularly molecular subtypes such as ER+/HER2+ and TNBC—which have distinct clinical behaviors and treatment responses.

## 7. Conclusions

Oligometastasis represents a paradigm shift in the management of metastatic cancer. Advances in imaging, local therapies, and systemic treatments have enabled more personalized approaches, improving the outcomes for patients with limited metastatic disease. Further research is essential to optimize treatment strategies and determine the biological underpinnings of this unique clinical entity. Evidence from trials such as EA2108 and MF07-01 underscores the complexity of integrating local and systemic approaches.

## Figures and Tables

**Table 1:** Ongoing prospective trials to confirm the prognostic efficacy of local therapy for breast cancer oligometastasis.

NCT Number	Study Title	Conditions	Interventions	Primary Outcome Measures	Phases	Enrollment	Start Date
NCT04698252	Local Therapy for ER/PR-positive Oligometastatic Breast Cancer (LARA)	Breast Cancer	Radiotherapy/surgery/radiofrequency ablation	Two-year progression-free survival (PFS): The PFS is defined as the time from randomization until the date of progression or death. The 2-year PFS rate represents the probability of a patient being free of progression 2 years after randomization and will be estimated using the Kaplan–Meier method from the baseline to 2 years later.	PHASE 2	74	1 April 2021
NCT03808337	Investigating the Effectiveness of Stereotactic Body Radiotherapy (SBRT) in Addition to Standard of Care Treatment for Cancer That Has Spread Beyond the Original Site of Disease	Triple-Negative Breast Cancer/Non-Small-Cell Lung Cancer	SBRT/systemic therapy/standard of care	Progression-free survival: To determine whether stereotactic body radiotherapy administered to all sites of metastatic disease in patients with oligometastatic non-small-cell lung cancer or triple-negative breast cancer improves progression-free survival (PFS), defined as time from randomization to disease progression or death, compared to standard-of-care therapy alone, for up to 2 years.	PHASE 2	145	16 January 2019
NCT03808662	Randomized Study of Stereotactic Body Radiation Therapy (SBRT) in Patients With Oligoprogressive Metastatic Cancers of the Breast and Lung	Triple-Negative Breast Cancer/Non-Small-Cell Lung Cancer	SBRT/standard of care	Progression-free survival: To study if the addition of early SBRT to the treatment of extra-cranial oligoprogressive metastatic disease could prolong the PFS compared to treatment with no SBRT. The PFS is defined as the time from randomization to disease progression or death and measured for up to 52 weeks after the final participant is enrolled.	PHASE 2	107	16 January 2019
NCT06135714	Metastasis-directed Therapy for Oligometastases of Breast Cancer (JCOG2110: ORIGAMI)	Breast Cancer	Systemic therapy for 12 weeks after primary registration/radiation therapy (SBRT/conventional RT) and surgery/same systemic therapy after secondary registration	Overall survival after second registration over 5 years.	PHASE 3	340	8 November 2023
NCT02089100	Trial of Superiority of Stereotactic Body Radiation Therapy in Patients With Breast Cancer (STEREO-SEIN)	Breast Cancer	SBRT/systemic treatment	Progression-free survival (PFS), events: Local recurrence, distant progression of target metastases, any new metastasis, death from any cause. Definition of progression is based on RECIST1.1 criteria. Progression is assessed locally, in any metastasis present at time of randomization or in any newly diagnosed metastasis, with minimal follow-up of 3 years for all patients.	PHASE 3	280	1 February 2014
NCT04646564	Radiotherapy for Extracranial Oligometastatic Breast Cancer	Breast Cancer	Standard of care/radiotherapy + standard of care	Progression-free survival: Time from randomization to disease progression at any site or death. Measured over 2 years.	PHASE 3	170	6 April 2021
